# Resistance of Feather-Associated Bacteria to Intermediate Levels of Ionizing Radiation near Chernobyl

**DOI:** 10.1038/srep22969

**Published:** 2016-03-15

**Authors:** Mario Xavier Ruiz-González, Gábor Árpád Czirják, Pierre Genevaux, Anders Pape Møller, Timothy Alexander Mousseau, Philipp Heeb

**Affiliations:** 1 Laboratoire Évolution et Diversité Biologique (EDB), UMR 5174 Centre National de la Recherche Scientifique (CNRS), Université Paul Sabatier, 118 Route de Narbonne, F-31062 Toulouse, France; 2 Department of Infectious Diseases, Faculty of Veterinary Medicine, University of Agricultural Sciences and Veterinary Medicine, Mănăştur street 3–5, RO-400372 Cluj-Napoca, Romania; 3 Department of Wildlife Diseases, Leibniz Institute for Zoo and Wildlife Research, Alfred-Kowalke-Straße 17, D-10315 Berlin, Germany; 4 Laboratoire de Microbiologie et de Génétique Moléculaires, Centre de Biologie Intégrative (CBI), Université de Toulouse, CNRS, UPS, France; 5 Laboratoire d’Ecologie, Systématique et Evolution, UMR 8079 CNRS, Université Paris-Sud, Bâtiment 362, F-91405 Orsay Cedex, France; 6 Department of Biological Sciences, University of South Carolina, SC 29208, USA

## Abstract

Ionizing radiation has been shown to produce negative effects on organisms, although little is known about its ecological and evolutionary effects. As a study model, we isolated bacteria associated with feathers from barn swallows *Hirundo rustica* from three study areas around Chernobyl differing in background ionizing radiation levels and one control study site in Denmark. Each bacterial community was exposed to four different γ radiation doses ranging from 0.46 to 3.96 kGy to test whether chronic exposure to radiation had selected for resistant bacterial strains. Experimental radiation duration had an increasingly overall negative effect on the survival of all bacterial communities. After exposure to γ radiation, bacteria isolated from the site with intermediate background radiation levels survived better and produced more colonies than the bacterial communities from other study sites with higher or lower background radiation levels. Long-term effects of radiation in natural populations might be an important selective pressure on traits of bacteria that facilitate survival in certain environments. Our findings indicate the importance of further studies to understand the proximate mechanisms acting to buffer the negative effects of ionizing radiation in natural populations.

Ionizing radiation is present all over the Earth. For more than four billion years, cosmic rays and the decay of radioactive isotopes have been two of the main sources of ionizing radiation^1^. Low levels of ionizing radiation found normally in nature are believed to be relatively benign for organisms^2^,^3^, although a recent review suggests otherwise^4^, and in humans radioactivity has been found to be associated with some types of cancers^5^. Exposure to ionizing radiation above certain levels (i.e. 1 Gy) has been demonstrated to have negative biological effects by damaging important biomolecules, such as DNA or proteins^6^. Novel and unnatural sources of ionizing radiation are of anthropogenic origin, the so called “nuclear and radiation accidents”, including events such as Three Mile Island (1979), Chernobyl (1986), and the Fukushima (2011) disasters, radioactive waste discharges (e.g. Mayak Complex at the Techa River, 1949–1952), or the testing of nuclear weaponry (more than 2000 tests have taken place since the Hiroshima bomb in 1945 and 32 nuclear weapons accidents have occurred^7^). The sudden release of large amounts of radionuclides has a direct impact on the health of the surrounding human population as well as ecological and evolutionary consequences^8^,^9^,^10^,^11^,^12^ because radioactive contamination effects on both soil and water sources can persist for decades to millennia at all trophic levels^13^. During the Chernobyl nuclear power plant disaster an area of 200,000 km^2^ was significantly polluted with 20 different radionuclides, and while many of these decayed within days, others such as ^137^Cs, ^90^Sr, and ^239^Pu and their decay products will be present for decades to millennia^12^.

The accidentally polluted areas at Chernobyl provide a suitable environment for studying the evolutionary effects that heterogeneous levels of radiation have on different organisms. At Chernobyl, a number of negative effects, i.e. chromosomal and cellular aberrations, and deleterious mutations, have appeared in many organisms (mammals, birds, insects, and plants, reviewed by Møller & Mousseau^11^). Moreover, a gradient in the ecological effects of radiation has been found, where places with high radiation dose showed low abundance and richness of birds, mammals and invertebrates^14^,^15^,^16^. Although lethal doses differ among taxonomic groups^9^; low levels of radiation increase the mutation rate of many organisms^17^,^18^,^19^,^20^. However, several microorganisms exhibit a wide range of tolerance to γ-radiation^4^,^21^,^22^ or even show a positive tropism towards radioactive substrates^23^. Although microorganisms play a key role in ecosystems, we know little about the effects of radiation on wild populations of microorganisms^24^. Increasing radiation was found to lead to a decline in diversity of fungi and bacteria^23^,^25^,^26^,^27^ (but see Ragon *et al.*^24^), and changes in microbial community composition^28^,^29^. Feather-associated bacterial assemblages comprises both environmental and host-specific bacteria, and an earlier study found that background environmental radiation had detrimental effects on the total cultivable bacterial communities^30^. The microbiota from radioactively polluted areas thus face new and strong selective pressures from background radiation, and microorganisms living in these areas can be expected to have evolved resistance towards radiation. Here we exposed bacterial communities isolated from the plumage of barn swallows, *Hirundo rustica*, sampled from nesting sites in areas close to Chernobyl with different background levels of radiation to different durations of experimental irradiation, and we examined their degrees of resistance to radiation exposure. Since barn swallows moult their feathers once annually between November and March^31^, all sampled feathers were ca. 5 months old, and most bacteria found on feathers must have colonized them from contaminated areas at Chernobyl, and Denmark.

## Results

### Bacterial isolates

We isolated 87 bacterial morphotypes: 18 from a high background radiation (2.9 μGy/h), 25 from an intermediate background radiation (0.45 μGy/h), 20 from a low background radiation (0.1 μGy/h), and 24 from a control study area with ambient background radiation (0.03 - 0.05 μGy/h). Sequencing of 16 S rDNA allowed us to identify 74 out of the 87 morphotypes to the species or genus level (listed in Supplementary Tables S1 and S2). Identified bacteria belonged mainly to the Bacillus-Firmicutes group (41 morphotypes) or the Actinobacteria (27), with low frequencies of γ-Proteobacteria (3), Flavobacteria (2), and β-Proteobacteria (1) (Supplementary Fig. S1). The lowest species richness was found at high background radiation regions.

### Effects of radiation on bacterial survival

To examine the effects of exposure to experimental radiation on bacterial survival, we analysed the mean numbers of colony forming units (CFUs) for bacterial morphotypes isolated respectively from high (14 morphotypes), intermediate (24), low (19), and control background radiation (22), which represents 67, 96.0, 95.0, and 96%, respectively, of the originally isolated morphotypes.

**Figure 1:**
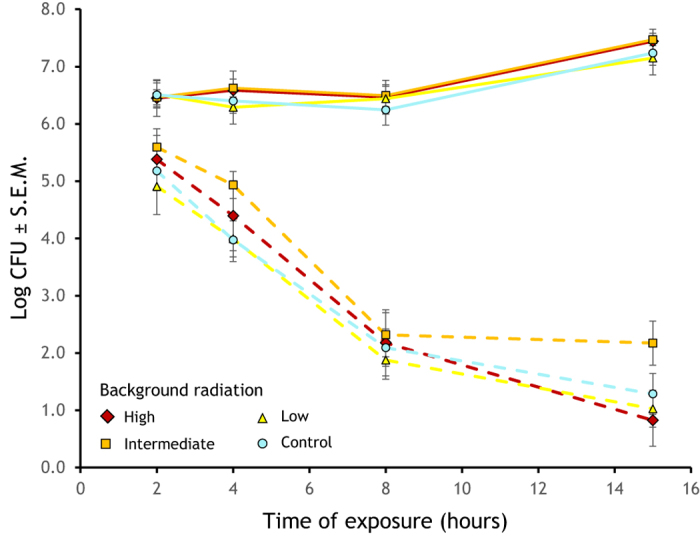
Number of Colony Forming Units of bacterial strains obtained from environments with different background radiation exposed to different radiation times (CFUs ± S.E.M.). Markers for background radiation in the environment are: high (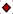), intermediate (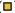), low (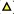), and control (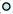). Samples not exposed to experimental radiation are plotted as continuous lines and experimentally exposed to radiation values are presented in dashed lines.

We found significant overall differences in survival for experimental exposure to radiation, time of exposure to experimental radiation (ANOVA, *F*_1,628_ = 913.962 and *F*_3,628_ = 44.361, respectively, *P* < 0.001) and intensity of background radiation (ANOVA, *F*_3,628_ = 4.846, *P* = 0.0024). There were significant effects for the interaction, experimental irradiation exposure × time of exposure (*F*_3,628_ = 91.008, *P* < 0.001; Fig. 1). Non parametric Kruskall-Wallis analyses for experimental exposure to radiation and duration of exposure produced similar results (*H* = 342.8 and *H* = 23.75, *P* < 0.0001 respectively) but not for background radiation (*H* = 2.926, *P* = 0.430; the ANOSIM analysis of the interactions of experimental exposure to radiation × time of exposure, and experimental exposure to radiation × background radiation, were significant (*R* = 0.61 and *R* = 0.26, *P* < 0.0001, respectively), but not the time of exposure × background radiation (*R* = 0.009, *P* = 0.26). The analyses of the geometric means produced similar results.

**Figure 2:**
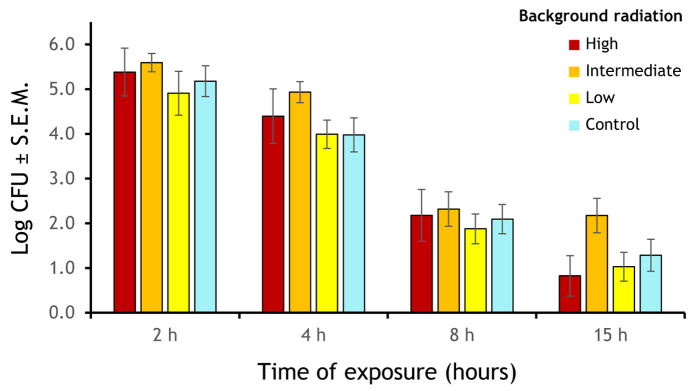
Population sizes of bacteria isolated from environments with different background radiation intensities exposed to four experimental radiation durations (log arithmetic means of CFUs + 1).

For the analysis of experimentally irradiated bacteria, we found significant overall effects for time of exposure (ANOVA, *F*_3,314_ = 91.99, *P* < 0.001) and background radiation (*F*_3,314_ = 4.405, *P* = 0.005) on the number of CFUs, but not for the interaction time of exposure × background radiation (*F*_9,314_ = 0.402, P = 0.94; Fig. 2).

### Effects of radiation on strains, phylogenetic group, and colony coloration

None of the bacterial strains in the treatments without experimental exposure to radiation went extinct, while 23.36% of the experimentally irradiated bacteria perished during the experiment (Fig. 1). Thus, we found a significant effect of experimental exposure to radiation treatment effect on strain survival (irradiated vs. not irradiated; *χ*^2^ = 144.77, d.f. = 1, *P* < 0.0001). There was a significant effect for duration of exposure to radiation on strain survival (*χ*^2^ = 54.92, d.f. = 3, *P* < 0.0001). For the experimentally irradiated bacteria, although mortality of strains from intermediate background radiation was lower (18.48%) than that of other background radiations (high, 28.85%; low, 26.32%; and control, 23.36%), the difference was not statistically significant (*χ*^2^ = 2.496, d.f. = 3, *P* = 0.48).

The main phylogenetic groups of bacteria in our samples were Actinobacteria and Bacillus – Firmicutes, as stated above (Supplementary Fig. S1). To test whether differences in the composition of species affected our results, we first compared Actinobacteria strains populations from different background radiations that were not exposed to experimental radiation. No significant differences were found: at 2 hours, *F*_3,24_ = 1.054, *P* = 0.389; at 4 hours, *F*_3,24_ = 2.301, *P* = 0.107; at 8 hours, *F*_3,24_ = 1.221, *P* = 0.327; and at 15 hours, *F*_3,24_ = 1.522, *P* = 0.238 (Supplementary Fig. S2). Then, we conducted the same tests on the community of Bacillus – Firmicutes species, and no significant differences were found: at 2 hours, *F*_3,35_ = 0.827, *P* = 0.489; at 4 hours, *F*_3,35_ = 0.448, *P* = 0.720; at 8 hours, *F*_3,35_ = 0.515, *P* = 0.675; and at 15 hours, *F*_3,35_ = 0.665, *P* = 0.579 (Supplementary Fig. S3). Thus, we did not detect significant differences in the phylogenetic composition of our most common samples.

**Figure 3:**
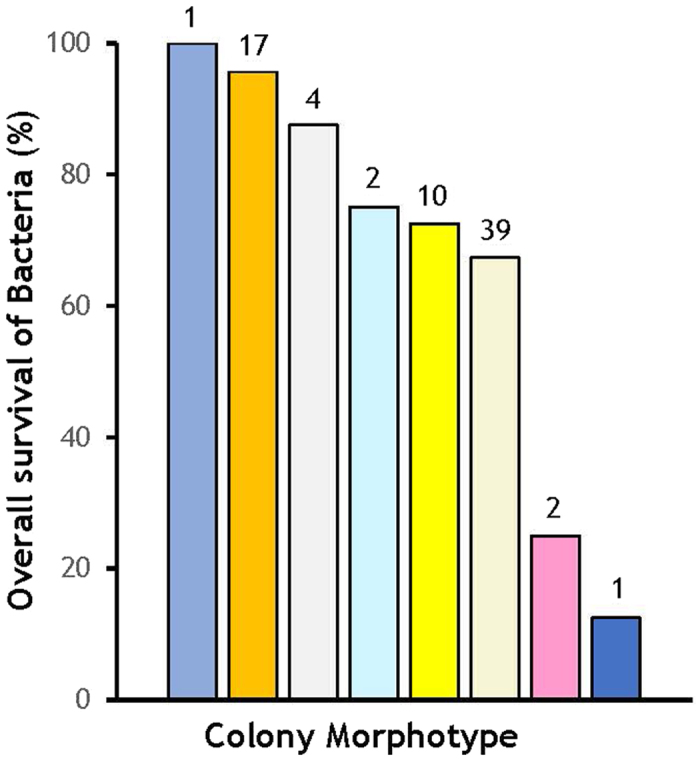
Overall survival of bacterial morphotypes grouped in categories based on colony colour or morphology. Numbers on top of bars represent the morphotypes grouped in each category (*n*): creamy (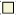), *Bacillus mycoides* (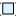), orange (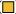), *Paenibacillus* sp2 (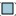), *Paenibacillus lactis* (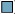), pink (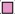), white (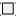), and yellow (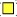).

The most common colony colorations were Cream, Orange, and Yellow (39, 17, and 10 samples, respectively; Fig. 3; Supplementary Fig. S4). We also found a few morphotypes that we allocated to other categories: White (Firmicutes, n = 4), Pink (Actinobacteria: *Microbacterium oleivorans* and *Williamsia* sp., n = 2), Feather-like colonies (*Bacillus mycoides*, n = 2), Shinny (*Paenibacillus* sp2, n = 1), and Swarming (*Paenibacillus lactis*, n = 1). We did not find significant differences in the numbers of CFUs produced when creamy, orange, and yellow bacterial species were not experimentally irradiated across different background radiation intensities (ANOVA, Supplementary Fig. S5–S7). There were significant overall differences in mortality among colonies of different coloration across all background radiations (*χ*^2^ = 28.5, d.f. = 7, *P* = 0.0002) and within each background radiation (high, χ^2^ = 17.52, d.f. = 3, *P* = 0.0006; intermediate, χ^2^ = 59.52, d.f. = 4, *P* < 0.0001; low, χ^2^ = 29.07, d.f. = 1, *P* < 0.0001; and, control, χ^2^ = 30.73, d.f. = 5, *P* < 0.0001). Among the three main colorations, Creamy morphotypes suffered the highest mortality (42.95%), which was similar to Yellow morphotypes (37.50%), whereas Orange morphotypes had the lowest mortality (8.82%; Fig. 3). Creamy morphotypes are more frequent in zones with lower background radiation, none and low (70%), while most of the Orange morphotypes were found at the intermediate background radiation area.

## Discussion

We have found that feather-associated bacterial communities from an intermediate background radiation site had lower mortality rates and were better able to tolerate exposure to four experimental doses of radiation than the bacterial communities from the other three study sites. In addition, this site hosted the largest number of orange pigmented bacteria, morphotypes that were found to be more resistant to radiation. The bacteria from the site with the highest background radiation had an intermediate survival ability when compared with the other sites. Our results show that intermediate background radiation may have selected for more resistant bacteria (Supplementary Fig. S8).

Three potential mechanisms could explain overall resistance to radiation of bacterial strains isolated from intermediate background radiation intensities: radiation might elicit induced responses; local microevolutionary responses; or long term evolution. Bacteria can resist radiation through different mechanisms such as the use of pigments (carotenoids) or the emergence of more efficient DNA or protein repair mechanisms^24^,^32^,^33^,^34^,^35^,^36^. Carotenoids are pigments that protect against oxidative stress and carotenoid-producing bacteria have been found to be associated with radioactive sites^37^. Unusually high DNA repair capacity has been found in the highly radio-resistant bacterium *Deinococcus radiodurans*^38^. Radio-resistance, however, seems strongly correlated with protein protection from oxidative damage, (e.g. by accumulating Mn^39^). Moreover, γ radiation induces the expression of certain heat shock proteins, such as GroES-GroEL, which are able to buffer against deleterious mutations by the proper folding of defective proteins^33^,^40^. A combination of these mechanisms may explain the performance of bacteria from the sites with intermediate background radiation levels. For example, the presence of orange pigmented bacteria might correlate with the presence of carotenoids that protect against oxidative damage induced by ionizing radiation. However, it might be expected that unpigmented morphotypes from these areas developed other protection mechanisms to minimise DNA or protein damage. The latter remains to be tested. On the other hand, low background ionizing radiation would have little evolutionary effect on the bacteria, thus, explaining why bacteria from areas with little radiation show similar resistance to radiation to the ones with no radiation or where the background radiation is typically very low. The bacteria that we tested have been surviving, multiplying, and evolving for more than 25 years in sites with three different background radiation levels that can be considered low-dose radiation, that is, less than 100 mGy and dose rates below 0.1 mGy min^−1^, e.g. ref. 41. Moreover, adaptive responses have been observed after doses of 1 to 500 mGy^35^, and effective DNA damage repair and defence pathways are activated with doses below 10 mGy^41^. Since bacteria lifespan ranges between less than an hour to several weeks, the bacteria from our sampling sites that, for example, survived for a month before cell division, received overall radiation doses ranging from 0.072 to 2.09 mGy. These doses are expected to trigger the molecular damage repair mechanisms, eliciting beneficial responses, and thus we should expect these populations to perform better when compared to the control population. Moreover, although we did find a higher resistance to radiation in the microbial community exposed to an intermediate background radiation dose of 0.45 μGy/h, the overall performance of this bacterial community is worse after irradiation than when it is not irradiated at all. A potential explanation for our findings is that while many studies have been made at the individual level, we are studying the effects of radiation at the population level across many generations. That is, low radiation doses might elicit a wide range of protective mechanisms at the individual level, thus resulting in the improvement of survival ability even if these responses entail cellular and molecular costs. But at the population level, the adaptive responses cannot be evaluated because we do not have data from the original populations prior to the start of the irradiation event caused by the Chernobyl accident for comparison of their performance. Thus, the results from our experiment might illustrate both the costs of an adaptive response and the negative effects of radiation. In addition, it might be possible that background radiation is not the only environmental factor driving the evolution of bacterial resistance to radiation, and thus selecting indirectly for bacteria that are more resistant to radiation. The later remains to be explored.

Finally, and as expected from previous findings^30^,^42^,^43^, we found that radiation had an overall negative effect on the survival of all bacteria, and that this effect increased with radiation dose. In conclusion, while little is known about the long-term effects of radiation in natural populations, the finding of bacterial resistance to intermediate levels of background radiation deserves further study to unveil the mechanisms adopted by these natural populations to endure the negative effects of low dose background radiation.

## Materials and Methods

### Ethical Statement

The research complied with requirements for research on birds in Ukraine and Denmark, and permission was given by the administration of the Chernobyl Exclusion Zone. All sampling was approved in an ethical review by the University of South Carolina Institutional Animal Care and Use Committee; the methods were carried out in accordance with the approved guidelines. All birds were handled briefly, and none died or showed signs of suffering during the short sampling period. All individuals flew upon release. The field studies did not involve endangered or protected species.

### Isolation of bacterial strains

In 2007 and 2008 wearing sterile gloves we collected eight feathers from the body of barn swallows randomly captured as part of an ongoing long-term project on birds breeding in Ukraine and Belarus^42^. Collections were made at collective farms in areas with high levels of radiation outside the southern exclusion zone near Chernobyl and following the procedure described by Czirják *et al.*^30^. All feathers were placed in a sealed plastic bag and stored at -20 °C in the dark until microbiological analysis. Host sex ratios were balanced (N_2007_ = 31 Females, 29 Males; N_2008_ = 58 Females, 56 Males). All farms presented similar climate and habitat types, surrounded by plantations, scattered trees, and open farmland, but were exposed to different environmental radiation levels^44^. The α, β, and γ radiation at ground level have previously been measured at each farm and cross-validated with measurements by the Ukrainian Ministry of Emergencies^45^. The two series of measurements were strongly positively correlated^30^,^44^. We chose bird samples from places with four different background radiation intensities: high, 2.9 μGy/h (Vesniane Farm, Ukraine); intermediate, 0.45 μGy/h (Farm 49, Vetka, Belarus); low, 0.1 μGy/h (Farm 43, Vetka, Belaryus); and, control, 0.03 - 0.05 μGy/h (Kraghede, Denmark).

In order to obtain both free-living and attached microorganisms, five feathers were sonicated at high frequency for 15 minutes in 3 repeats (5 minutes each with a 5-minute pause between repeats) in 0.8 ml of sterile physiological (0.90% w/v) saline solution for bacterial declumping. This process does not affect bacterial viability^46^. After sonication samples were vortexed for 20 seconds and bacterial suspensions were transferred to a sterile 1.5 ml eppendorf tube. Then, feathers were re-suspended in 0.5 ml sterile physiological saline and vortexed again for 20 seconds. The supernatant was transferred to a second sterile eppendorf, obtaining an overall volume of ~1.3 ml solution. The method is fully described by Czirják *et al.*^30^. All samples were treated in the same way, and thus, any potential bias would have affected bacterial communities equally.

In duplicate, we spread 100 μl each of the microbial solutions on Tryptic Soy Agar (TSA, #22091, Fluka), a rich growth medium, and the plates were incubated at 25 °C, for 3 days. To inhibit fungal growth we added 100 mg mL^−1^ of cycloheximide (#01810, Fluka) to the medium. Then, we selected all bacterial colonies showing morphological differences based on colour, shape, and size, for all the sixteen plates per site, and we inoculated tubes containing 15 mL of Tryptic Soy Broth (TSB, #22092, Fluka) medium with single colonies until cultures reached an optical density of 0.6 at a wavelength of 600 nm. The tubes were centrifuged and the pellets re-suspended in 10 mL of TSB.

### Irradiation

Bacterial morphotypes isolated as above were randomly allocated across the wells of a 96-well microtiter flat plate (84 isolates and three replicates of an *Escherichia coli* K-12 W3110 laboratory strain as control). We used 15 μl of each culture to inoculate 135 μl of TSB per well. The same inoculation order was kept to culture 16 microtiter plates that were allocated to radiation and no radiation treatments with a replicate each. Thus, each bacterial isolate had two replicates and the control *E. coli* six replicates for each treatment. Three samples did not grow well in TSB medium during the experiment and they were removed from the experiments (two from high and one from low background radiation respectively).

Samples were exposed to four different doses of radiation with a BIOBEAM 8000 γ irradiator (IFR 31, Service de Médicine Nucléaire de Rangueil, Hôpital de Rangueil, Toulouse, France) equipped with a ^137^Cs source to deliver radiation at a dose rate of 3.82–4.4 Gy/min: 2-hours exposure, 0.458–0.528 kGy; 4 hours, 0.917–1.056 kGy; 8 hours, 1.833–2.112 kGy; and, 15 hours, 3.438–3.960 kGy. Control plates (No radiation) were not exposed, but placed close to the irradiator in the same room for the same period of time as the radiation treatment plates to ensure similar environmental conditions.

Immediately after the treatment the corresponding plates were kept at 4 °C. Five μL of each individual culture were serially diluted into a new microtiter plate containing 195 μL of TSB. For each sample five μL of each dilution were spotted on LB agar plates to calculate the number of C.F.U.s per mL after 24 h of incubation at 37 °C. Overall bacterial populations were calculated by multiplying the number of CFUs for the corresponding dilution factor. Five samples were removed from all the analysis and three more were excluded from the non-parametric pairwise comparisons (Supplementary Table S3).

### Microbial species identification

The 16 S rDNA was amplified with the universal primers fD1 and rP2^47^. The PCR products were sequenced in 2012 to identify the microbial species when possible and sequences are deposited in GenBank (Supplementary Table S1).

### Statistical analyses

We calculated both the geometric and the arithmetic means of the two counts of the number of CFUs for each bacterial morphotype, the former to lessen the impact of dissimilar values on meaningful statistical analyses and the latter because under radiation one of the CFUs counts was zero in few cases. All analyses shown in the text were done using the arithmetic mean except mentioned otherwise. The means were transformed using log (*y* + 1) to approximate a normal distribution. We further recorded the mortality of strains and we used a *χ*^2^-test to examine differences. We used a three-way ANOVA to examine overall differences in the number of CFUs produced by each bacterial morphotype with experimental exposure to radiation (irradiated vs. no irradiated), time of exposure (2, 4, 8, and 15 hours), and background radiation (high, intermediate, low, and control) as factors. ANOVAs were performed with the R software^48^.

Due to the nature of the observations, however, our transformed data did not meet the assumption of homoscedasticity in many of the analysis. Therefore, we conducted Kruskall-Wallis tests to test for overall differences in bacteria survival for experimental exposure to radiation, time of exposure, and background radiation intensity; and post hoc Mann-Whitney pairwise comparisons within each factor, Bonferroni corrected. Then, we calculated 1-way ANOSIM^49^, using Bray-Curtis distance, to test for the interactions between background radiation intensity and treatment, treatment and time of exposure, and background radiation intensity and time of exposure. We estimated both sequential Bonferroni significance and corrected Bonferroni *P*-values. We used the free software PAST^50^.

To examine differences in the performance of each community of bacteria isolated from sites with different background radiation intensity within the experimental exposure to radiation treatment, we used a two-way ANOVA with time of exposure (2, 4, 8, and 15 hours) as and background radiation (high, intermediate, low, and control) as factors.

Finally, we explored whether colony colour had an effect on bacterial mortality under irradiation conditions. To do so, first, we visually allocated bacterial morphotypes into eight different categories, established beforehand by the authors, that are based on colony colour or morphology by sight: Creamy (all those beige, pale yellow or slightly pink, and creamy colonies), Orange (all orange, peach or pale orange or peach colonies), *Paenibacillus* sp1 (big pale chiral pattern-forming bacteria), *Paenibacillus* sp2 (translucent swarming bacteria), Pink (pink colonies), Shiny (white and very lustrous colonies), White (white colonies), and Yellow (yellow colonies). Then, we quantified the overall mortality across treatments and places for each bacterial morphotype. We used a *χ*^2^-test to examine differences across background radiation intensity, experimental exposure to radiation, and single bacteria morphotypes across treatments when the morphotype was represented by at least 10 colonies.

## Additional Information

**How to cite this article**: Ruiz-González, M. X. *et al.* Resistance of Feather-Associated Bacteria to Intermediate Levels of Ionizing Radiation near Chernobyl. *Sci. Rep.* **6**, 22969; doi: 10.1038/srep22969 (2016).

## Supplementary Material

Supplementary Information
