# Supplementary material for: Resistance of Feather-Associated Bacteria to Intermediate Levels of Ionizing Radiation near Chernobyl
Source: Sci Rep. 2016 Mar 15;6:22969. doi: 10.1038/srep22969 (PMC4792135; doi:10.1038/srep22969)
Supplement: Supplementary Information [file srep22969-s1.pdf]

SUPPLEMENTARY INFORMATION

"Resistance of Feather-Associated Bacteria to Intermediate Levels of Ionizing Radiation near Chernobyl"

Mario Xavier Ruiz-González, Gábor Árpád Czirják, Pierre Genevaux, Anders Pape Møller, Timothy A. Mousseau and Philipp Heeb

CONTENTS

SUPPLEMENTARY SUPPORTING DATA

- **Supplementary Table S1. Genbank accession numbers.**
- **Supplementary Table S2. Bacterial taxa found at different background radiation intensities after 16S rDNA sequencing.**
- **Supplementary Figure S1. Bacterial phylogenetic groups and number of species identified after 16S rDNA sequencing found at different background radiation intensities near Chernobyl and Denmark.**
- **Supplementary Figure S2. Actinobacteria species isolated from different background radiation.**
- **Supplementary Figure S3. Bacillus - Firmicutes species isolated from different background radiation.**
- **Supplementary Figure S4. Bacteria colonies morphotypes.**
- **Supplementary Figure S5. Cream coloured bacteria from different background radiation.**
- **Supplementary Figure S6. Orange coloured bacteria from different background radiation.**

- **Supplementary Figure S7. Yellow coloured bacteria from different background radiation.**
- **Supplementary Figure S8. Species from different background radiation facing new exposure to radiation.**
- **Supplementary Table S3. Raw data.**

SUPPLEMENTARY SUPPORTING DATA

Supplementary Table S1. **Genbank Accession Numbers.** A: High background radiation; B: Intermediate background radiation; C: low background radiation; D: control ambient radiation; W: control *E. coli* strains.

Accession Number	ID	Length	Sequence Match to
KT382372	A11	1432	*Staphylococcus hominis* strain N7
KT382373	A14	1435	*Sporosarcina aquimarina* strain 38
KT382374	A15	1414	*Curtobacterium herbarum* strain P 420/07
KT382375	A16	1397	*Moraxella* sp. BQEN3-02
KT382376	A17	1435	*Paenibacillus* sp. Enf35
KT382377	A18	1433	*Staphylococcus pasteuri* strain BQN3B-03d
KT382378	A1	1422	*Staphylococcus aureus* subsp. *aureus* ST228
KT382379	A2	1401	*Microbacterium* sp. 11.2 AW
KT382380	A3	844	*Rhodococcus erythropolis* strain T285
KT382381	A5	1392	*Dietzia* sp. II_Gauze_W_12-11
KT382382	A6	1397	*Williamsia* sp. L1505
	A7	163	*Bacillus pumilus* strain AIMST Lce44
KT382383	A9	1394	*Pseudoclavibacter helvolus* strain CJ-G-TSA2
KT382384	B10	1403	*Micrococcus* sp. HaNA19
KT382385	B11	1167	*Bacillus pumilus* strain LZBP-10
KT382386	B12	773	*Paenibacillus tundrae* strain E8a
KT382387	B13	1415	*Staphylococcus sciuri* strain R1-4A
KT382388	B14	1440	*Enterococcus* sp. HAMBI3063

KT382389	B15	1406	*Citricoccus alkalitolerans* strain YIM 70010
KT382390	B16	1412	*Brevibacterium* sp. B-1082
KT382391	B17	1400	*Citricoccus* sp. KMM 3890
	B18		Amplification Failed Twice
KT382392	B19	1220	*Sejongia* sp. 5516J-09
	B1		Amplification Failed Twice
KT382393	B20	1396	*Janibacter anophelis* strain CCUG 49715
KT382394	B22	838	*Bacillus pumilus* strain LZBP-10
KT382395	B23	1399	*Staphylococcus aureus* subsp. *aureus* ST228
KT382396	B24	1446	Virgibacillus sp. WS 4627
KT382397	B25	1424	*Bacillus mycoides*
KT382398	B2	1315	*Kocuria* sp. RV89
	B3		Amplification Failed Twice
KT382399	B4	1432	*Staphylococcus haemolyticus* strain BQN1L-01d
KT382400	B5	1393	*Brachybacterium arcticum* strain Lact 5.2
KT382401	B6	1430	*Staphylococcus* sp. BQN4T-04
KT382402	B7	1401	*Sejongia* sp. 5516J-09
KT382403	B8	1432	*Staphylococcus vitulinus* strain ATCC 51145
KT382404	B9	1430	*Bacillus licheniformis* strain LZBL-9
KT382405	C10	1403	*Mycetocola* sp. PX8c_S1
KT382406	C11	1431	*Bacillus clausii* strain BG-B15
KT382407	C12	1428	*Bacillus pumilus* strain LZBP-10
KT382408	C13	1423	*Escherichia coli* O83:H1 str. NRG 857C
KT382409	C14	1409	*Citrococcus* sp. ITM10
KT382410	C15	1427	*Escherichia coli* strain BE27

	C16		Amplification Failed Twice
KT382411	C17	1405	*Janibacter* sp. HR08-44
KT382412	C18	1439	*Staphylococcus saprophyticus* strain CTSPL7
KT382413	C19	1405	*Janibacter* sp. HR08-44
KT382414	C1	1162	*Staphylococcus aureus* M1
KT382415	C20	1431	*Staphylococcus epidermidis* strain CIFRI H-TSB-11-ZMA
KT382416	C2	1409	*Brevibacterium* sp. EP11
KT382417	C3	1412	*Brevibacterium* sp. B-1082
KT382418	C4	1436	*Bacillus* sp. QT14
KT382419	C5	1443	*Staphylococcus aureus* strain MSSA476
KT382420	C6	1424	*Bacillus pumilus* strain CHRPB37
KT382421	C7	1404	*Citricoccus alkalitolerans* strain YIM 70010
KT382422	C8	1400	*Dietzia* sp. TmT3-14-1
KT382423	C9	1398	*Janibacter* sp. TMB2-14
KT382424	D10	1403	*Arthrobacter rhombi* strain HR103
KT382425	D11	1432	*Bacillus licheniformis* strain DQgbc4
KT382426	D12	1435	*Bacillus megaterium* strain XAS4-7
KT382427	D13	1437	*Paenibacillus* sp. Enf35
KT382428	D14	1432	*Sporosarcina* sp. SS6.9
KT382429	D15	1436	*Paenibacillus lactis* strain MB 1871
KT382430	D16	1429	*Bacillus aerophilus* strain KUDC1741
KT382431	D17	698	*Staphylococcus aureus* strain GiTSA
	D18		Amplification Failed Twice
KT382432	D19	1431	*Bacillus licheniformis* strain LZBL-3

KT382433	D1	758	*Bacillus mycoides* strain MR-R4
KT382434	D20	1411	Uncultured soil bacterium clone PK_III
KT382435	D21	1211	*Bacillus pumilus* strain LZBP-10
KT382436	D2	1438	*Virgibacillus* sp. NOT2
KT382437	D22	1406	*Microbacterium* sp. 11.2 AW
KT382438	D24	1450	*Virgibacillus* sp. WS 4627
KT382439	D3	296	*Staphylococcus aureus* strain X13
KT382440	D4	424	*Bacillus pumilus* strain Bp1
KT382441	D6	1146	*Rhodococcus fascians* strain KSI 126
KT382442	D7	1398	*Arthrobacter* sp. KA2-29
KT382443	D8	1328	*Bacillus* sp. strain FR-W2C1
KT382444	D9	1403	*Microbacterium oleivorans* strain DSM 16091
	W3.1	1424	*Escherichia coli*
	W3	1422	*Escherichia coli*
	W3	1415	*Escherichia coli*

Supplementary Table S2. Bacterial taxa found at different background radiation intensities after 16S rDNA sequencing. The raw sequences were compared to the NCBI nucleotide databases using blastn.

**Taxonomic Group**	**Taxon**	**High**<br>2.9 µGy/h	**Intermediate**<br>0.45 µGy/h	**Low**<br>0.1 µGy/h	**Control**<br>0.03 - 0.05 µGy/h
Actinobacteria	*Arthrobacter rhombi*				1
	Arthrobacter sp.				1
	Brachybacterium sp.		1		
	Brevibacterium sp. 1		1	1	
	Brevibacterium sp. 2			1	
	Citrococcus sp. 1			1	
	Citrococcus sp. 2		2	1	
	Curtobacterium herbarum	1			
	Dietzia sp. 1	1			
	Dietzia sp. 2			1	
	Janibacter sp. 1		1		
	Janibacter sp. 2			1	
	Janibacter sp. 3			2	
	Kokuria sp.		1		
	Microbacterium oleivorans				1
	Microbacterium sp.				1
	Micrococcus sp.		1		
	Mycetocola sp.			1	
	Pseudoclavibacter sp.	1			

	Rhodococcus sp. 1				1
	Rhodococcus sp. 2		1		
	Williamsia sp.	1			
Bacillus-Firmicutes	*Bacillus aerophilus*				1
	Bacillus clausii			1	
	Bacillus licheniformis st. 1		1		1
	Bacillus licheniformis st. 2				1
	Bacillus megaterium				1
	Bacillus mycoides		1		1
	Bacillus pumilus st. 1	1	2	1	2
	Bacillus pumilus st. 2			1	
	Bacillus sp. 1				
	Bacillus sp. 2			1	1
	Enterococcus sp.		1		
	Paenibacillus lactis				1
	Paenibacillus sp. 1	1			1
	Paenibacillus sp. 2		1		
	Sporosarcina aquimarina	1			
	Sporosarcina sp.				1
	Staphylococcus aureus st. 1	1	1	1	2
	Staphylococcus aureus st. 2			1	
	Staphylococcus epidermis			1	
	Staphylococcus haemolyticus		1		
	Staphylococcus hominis	1			

	Staphylococcus pasteuri		1			
	Staphylococcus saprophyticus			1	1	
	Staphylococcus sciuri			1		
	Staphylococcus vitulinus			1		
	Virgibacillus sp. 1			1		1
	Virgibacillus sp. 2					1
β-Proteobacteria	*Pigmentiphaga* sp.					1
γ-Proteobacteria	*Escherichia coli* st. 1				1	
	Escherichia coli st. 2				1	
	Moraxella sp.		1			
Flavobacteria	*Sejongia* sp.			2		
**Actinobacteria**		27	6	7	9	5
**Bacillus-Firmicutes**		41	6	12	8	15
**β-Proteobacteria**		1	0	0	0	1
**γ-Proteobacteria**		3	1	0	2	0
**Flavobacteria**		2	0	2	0	0
		74	13	21	19	21

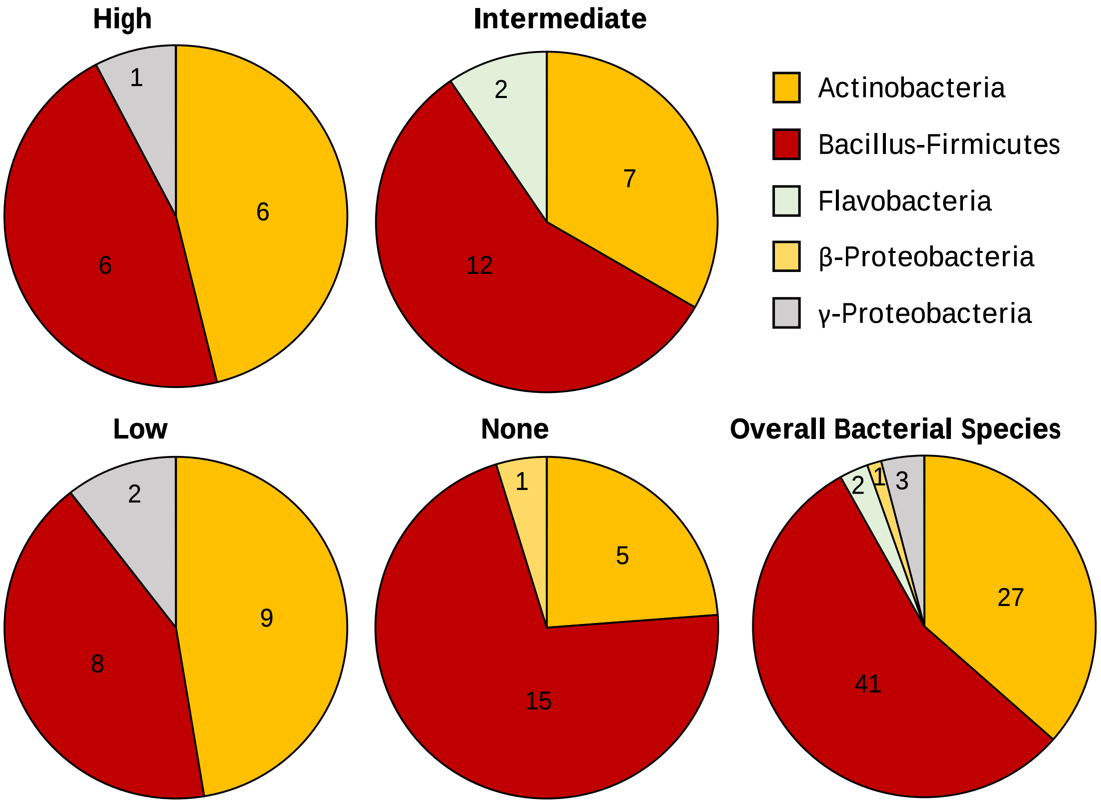

Supplementary Figure S1. Bacterial phylogenetic groups and number of species identified after 16S rDNA sequencing found at different background radiation intensities near Chernobyl and Denmark.

Supplementary Figure S2. Population sizes (CFUs) of Actinobacteria species isolated from different background radiation: high (5 spp.), intermediate (7 spp.), low (8 spp.), and control (5 spp.). For each colour, first bars on the left are the no irradiated control and the second bar represents the radiation treatment.

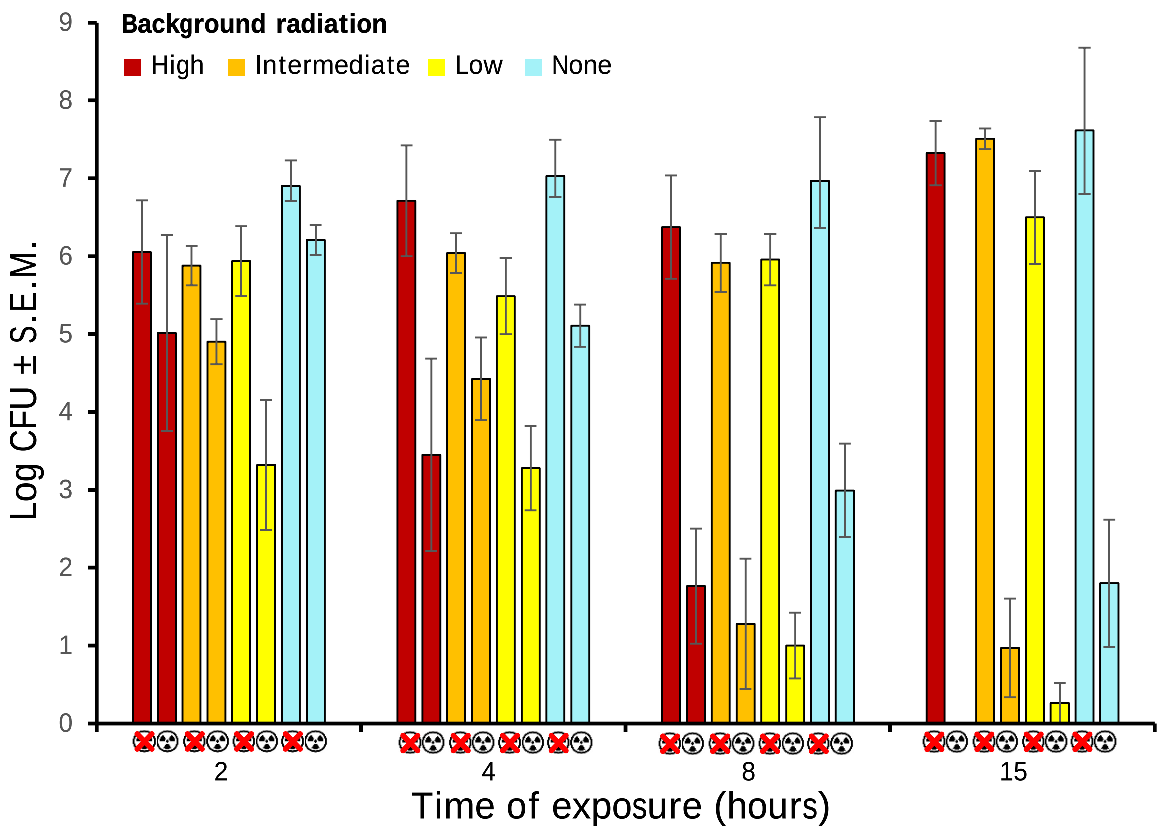

Supplementary Figure S3. Population sizes (CFUs) of Bacillus - Firmicutes species isolated from different background radiation: high (4 spp.), intermediate (11 spp.), low (8 spp.), and control (13 spp.). First bars are the no irradiated control and each second bar represents the radiation treatment.

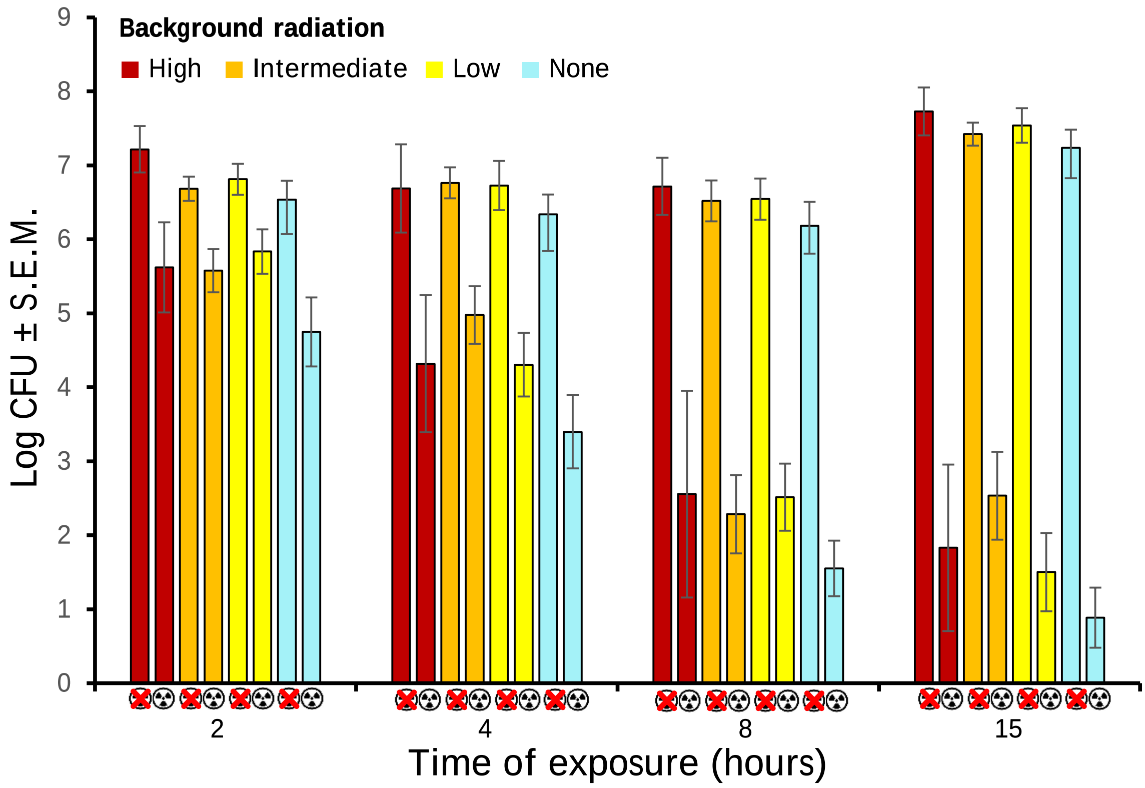

Supplementary Figure S4. Examples of bacterial colony morphotypes. Species were identified after 16S sequencing. Scale indicates 0.5 cm.

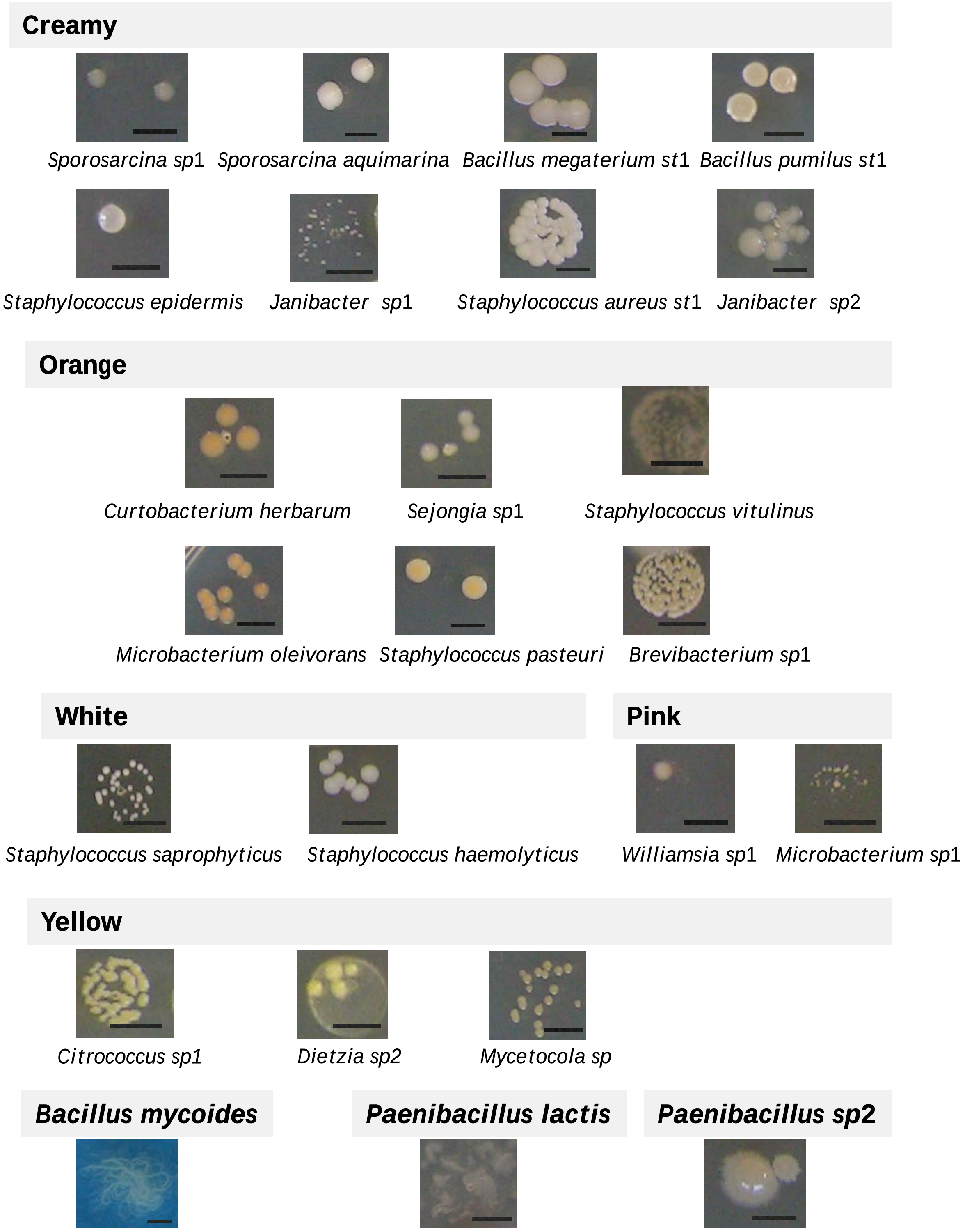

Supplementary Figure S5. Cream coloured bacteria isolated from different background radiation: high (6 spp.), intermediate (6 spp.), low (15 spp.), and control (12 spp.). First bars are the control and each second bar represents the radiation treatment. Control cream coloured bacteria populations from different background radiations and not exposed to experimental radiation (first bars for each background radiation) were compared with one-way ANOVAs for each time exposure. No significant differences were found (at 2 hours, $F_{3,38}$ = 0.112, P = 0.952; at 4 hours, $F_{3,38}$ = 0.046, P = 0.987; at 8 hours, $F_{3,38}$ = 0.009, P = 0.999; and at 15 hours, $F_{3,38}$ = 0.194, P = 0.899; data not shown).

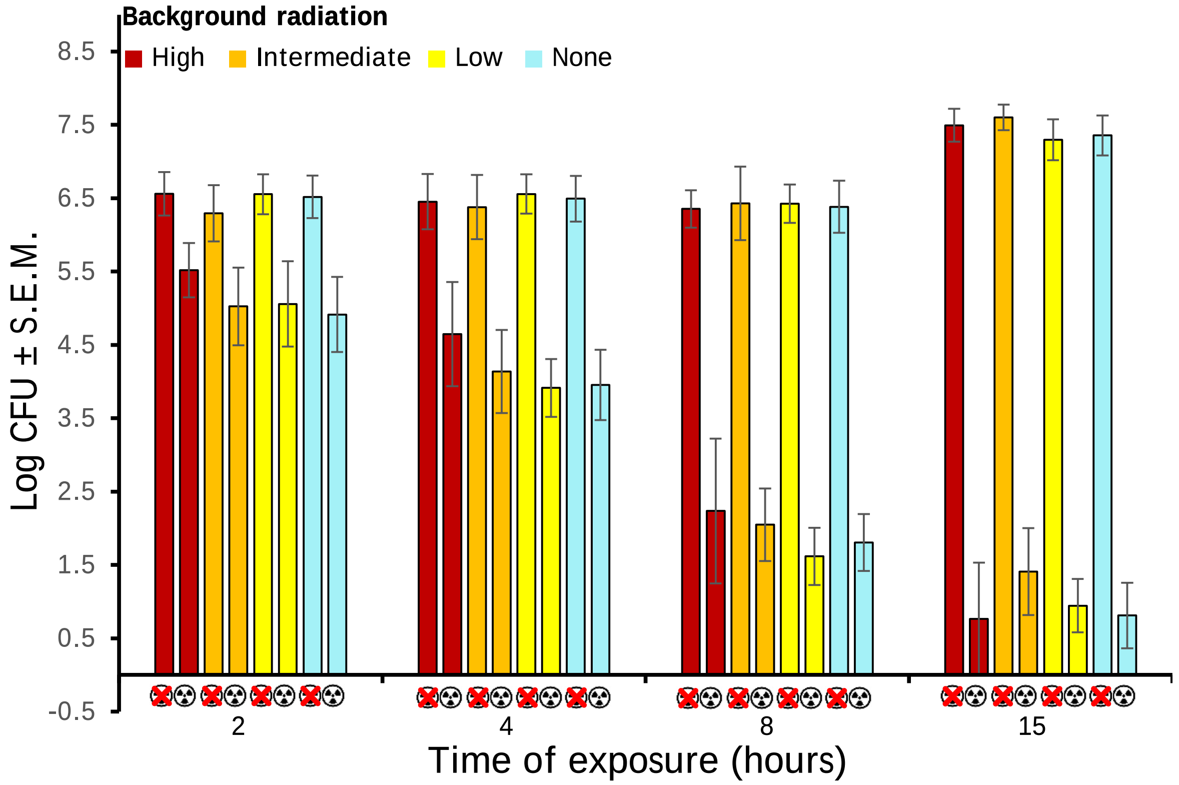

Supplementary Figure S6. Orange coloured bacteria isolated from different background radiation: high (3 spp.), intermediate (10 spp.), low (1 sp.), and control (3 spp.). First bars are the control and each second bar represents the radiation treatment. Control orange coloured bacteria populations from different background radiations and not exposed to experimental radiation (first bars for each background radiation) were compared with one-way ANOVAs for each time exposure. No significant differences were found (at 2 hours, $F_{3,16}$ = 0.198, P = 0.896; at 4 hours, $F_{3,16}$ = 0.206, P = 0.890; at 8 hours, $F_{3,16}$ = 0.217, P = 0.883; and at 15 hours, $F_{3,16}$ = 0.109, P = 0.953; data not shown).

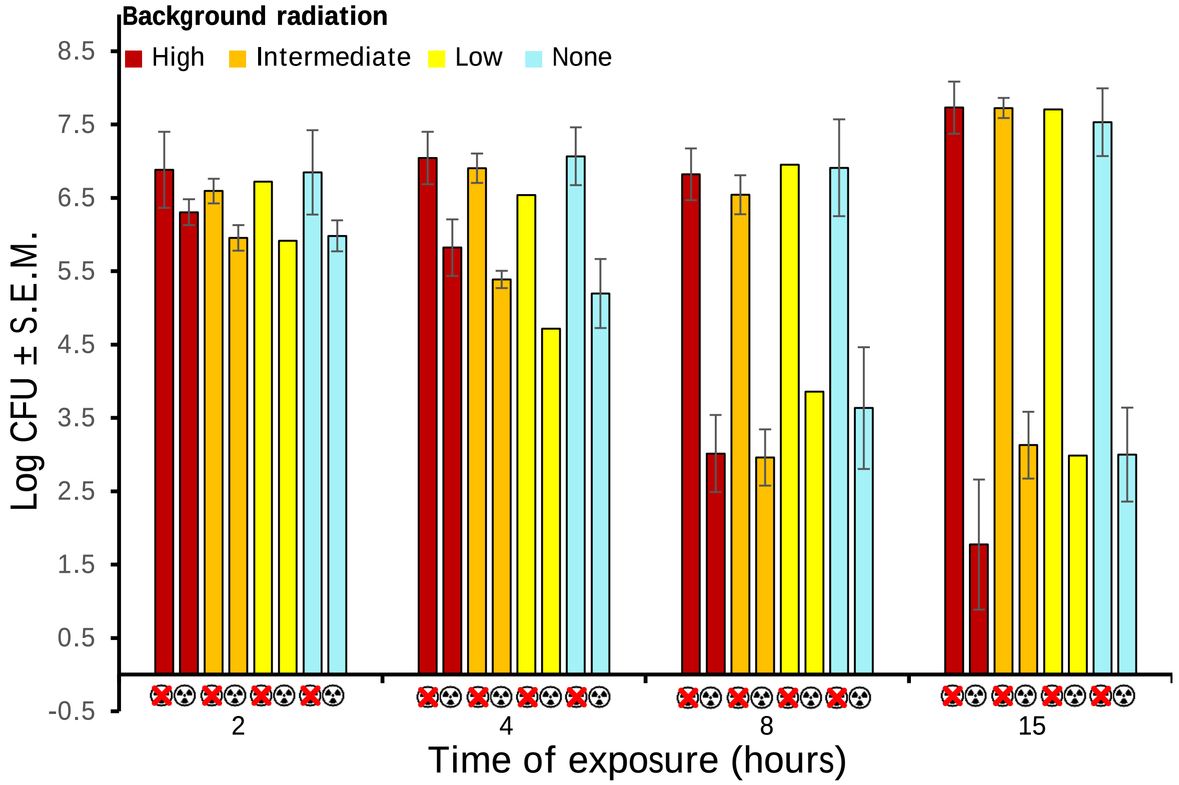

Supplementary Figure S7. Yellow coloured bacteria isolated from different background radiation: high (1 sp.), intermediate (4 spp.), low (3 spp.), and control (2 spp.). First bars are the control and each second bar represents the radiation treatment. Control yellow coloured bacteria populations from different background radiations and not exposed to experimental radiation (first bars for each background radiation) were compared with one-way ANOVAs for each time exposure. No significant differences were found (at 2 hours, $F_{3,9} = 0.378$, $P = 0.773$; at 4 hours, $F_{3,9} = 1.300$, $P = 0.358$; at 8 hours, $F_{3,9} = 1.015$, $P = 0.449$; and at 15 hours, $F_{3,9} = 0.900$, $P = 0.494$; data not shown).

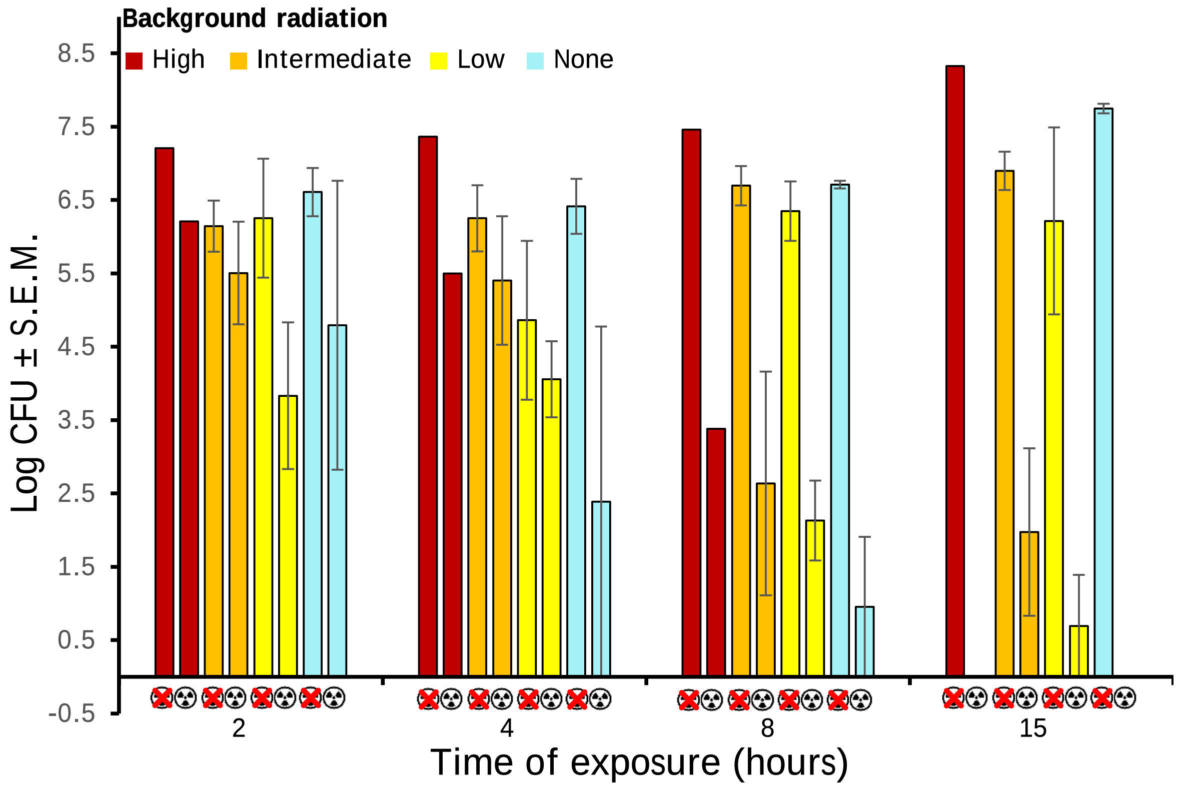

Supplementary Figure S8. Species from different background radiation facing new exposure to radiation. Bars represent mortality and resistance of bacterial species from different radiation backgrounds: high radiation (11 spp.), intermediate radiation (23 spp.), low radiation (18 spp.), and no radiation control (19 spp.).

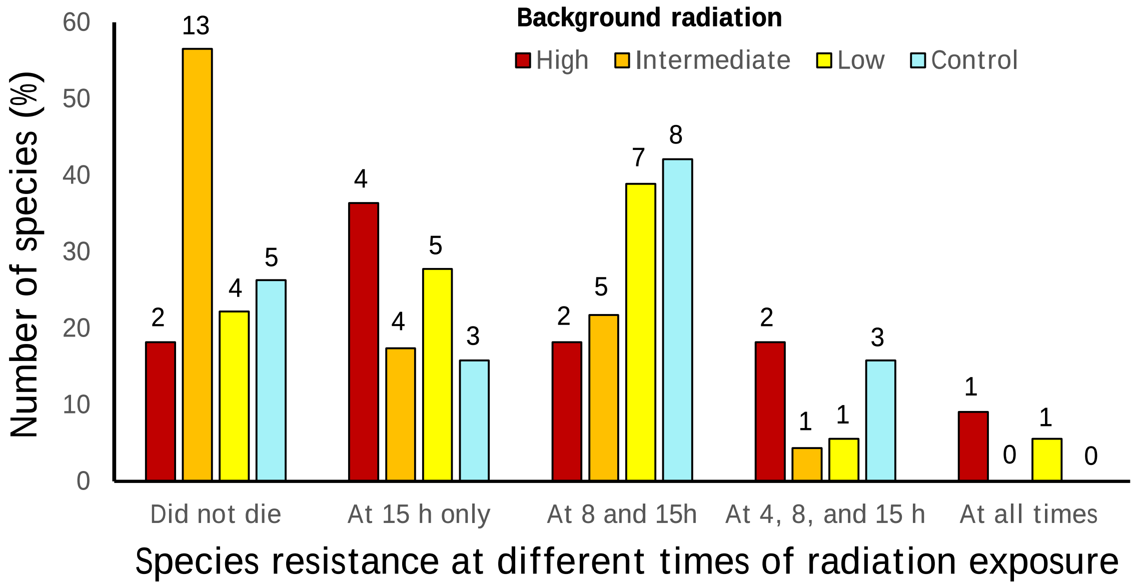

Supplementary Table 3. C.F.U.s counts. **Well** column denotes the position of each bacterial sample in the microtiter plates. **ID** is the identification code for each bacterium (A: High background radiation - Vesniane; B: Intermediate background radiation - Farm 49; C: low background radiation - Farm 43; D: ambient control background radiation - Denmark; W: *E. coli*). **Dilut.** is the dilution (1: undiluted; 2: 40 times diluted; 3-8: 10.75 times diluted at each spot). **C** denotes bacteria not exposed to experimental radiation and **R** experimental exposure to radiation. T2, T4, T8, and T15 are the time of exposure in hours. Samples removed from all analysis: A5, A11, B24, C17, and D24. And A7, A16, and D21 were excluded from the non-parametric pairwise comparisons

**Well**	**ID**	**Dilution**	**C T2**	**Dilution**	**R T2**	**Dilution**	**C T4**	**Dilution**	**R T4**	**Dilution**	**C T8**	**Dilution**	**R T8**	**Dilution**	**C T15**	**Dilution**	**R T15**
F11	A1	7	2	5	41	7	8	3	1	6	1	2	2	7	24	0	0
F11	A1	8	2	6	5	8	1	4	20	7	6	0	0	8	5	0	0
H3	A10	5	10	5	11	5	1	5	4	5	1	2	7	6	5	0	0
H3	A10	6	3	6	1	6	4	6	1	6	3	3	3	8	2	0	0
D5	A11	0	0	0	0	2	1	0	0	0	0	4	16	0	0	2	1
D5	A11	0	0	0	0	0	0	0	0	0	0	5	1	0	0	3	1
C5	A14	6	5	5	5	6	7	5	10	6	4	6	2	7	3	3	1
C5	A14	7	2	6	3	7	2	6	1	7	1	7	1	8	1	4	17
A3	A15	6	15	6	4	7	3	6	3	6	23	2	26	7	20	0	0
A3	A15	7	3	7	1	8	1	7	1	7	1	3	3	8	2	0	0
E5	A16			2	1	4	18	3	19	4	9	2	1	5	14	0	0
E5	A16			3	13	5	3	4	3	5	3			6	2	0	0
A2	A17	6	2	3	9	4	9	2	2	5	8	0	0	6	7	0	0
A2	A17	7	1	4	2	5	3			6	1	0	0	7	2	0	0
C4	A18	7	5	5	32	6	22	4	29	6	31	2	6	7	11	2	5
C4	A18	8	1	6	5	7	3	5	5	7	5			8	3	3	2
G1	A2	6	3	5	14	7	4	2	1	7	2	0	0	6	11	0	0
G1	A2	7	1	6	7	8	2			8	1	0	0	7	1	0	0
F5	A3	5	14	5	1	5	10	3	1	5	9	2	1	6	24	0	0
F5	A3	6	1	6	3	7	2	4	3	6	2	3	1	7	6	0	0
H1	A4	5	23	4	1	5	28	5	4	6	3	0	0	6	13	0	0
H1	A4	6	6	5	8	6	5	7	1	7	2	0	0	7	2	0	0
G3	A5	0	0	0	0	0	0	0	0	0	0	0	0	6	5	0	0
G3	A5	0	0	0	0	0	0	0	0	0	0	0	0	7	4	0	0
C3	A6	3	8	0	0	3	20	0	0	3	15	0	0	5	5	0	0

C3	A6	4	1	0	0	4	2	0	0	4	3	0	0	6	4	0	0
F3	A7	8	1			7	3	5	1	7	4	3	3	8	2	0	0
F3	A7	7	3			6	14	4	25	6	16	2	13	7	6	2	6
B3	A8	5	21	5	18	5	41	5	6	5	25	3	15	6	18	2	9
B3	A8	6	1	6	2	6	5	6	1	6	3	4	2	7	2	3	1
F10	A9	6	28	5	1	6	44	5	2	6	1	2	5	7	20	0	0
F10	A9	7	3	6	6	7	4	6	1	7	10	4	1	8	5	0	0
G2	B1	6	4	5	5	5	1	4	5	6	11	2	1	7	4	3	9
G2	B1	7	2	6	1	6	11	5	3	7	4	3	4	8	2	4	1
G11	B10	5	8	5	12	5	38	6	2	5	27	4	27	7	2	3	13
G11	B10	6	2	6	1	6	1	7	1	6	11	5	2	8	1	4	2
F6	B11	5	1	5	1	7	4	4	11	6	14	2	8	7	5	4	1
F6	B11	6	9	6	1	8	1	5	5	7	2	3	3	8	1	6	1
B12	B12	6	2	4	1	6	1	4	1	6	1	2	1	7	1	2	1
B12	B12	7	1	5	1	7	1	5	1	7	1	3	1	8	1	3	1
A8	B13	7	1	5	18	5	1	4	36	5	39	2	17	7	9	3	15
A8	B13	8	1	6	3	6	13	5	4	6	8	3	2	8	1	4	5
E12	B14	6	10	4	1	5	56	5	42	6	11	0	0	5	1	3	18
E12	B14	7	1	5	6	6	9	6	7	7	1	0	0	7	1	4	3
A4	B15	6	22	4	30	5	1	2	1	6	1	0	0	6	10	0	0
A4	B15	7	2	5	1	6	13	3	11	7	1	0	0	7	3	0	0
B8	B16	5	26	4	1	6	2	4	29	5	56	0	0	7	9	0	0
B8	B16	6	1	5	20	7	2	5	4	6	7	0	0	8	1	0	0
D1	B17	4	30	3	1	4	1	2	1	5	6	0	0	6	12	0	0
D1	B17	5	5	4	3	5	5	3	3	6	4	0	0	7	3	0	0
D4	B18	6	8	6	3	6	14	4	1	7	1	2	14	6	20	3	6
D4	B18	7	2	7	2	7	1	5	6	8	1	3	3	7	5	4	1
G8	B19	6	9	5	11	6	12	4	13	6	4	2	1	6	1	3	9
G8	B19	7	1	6	1	8	1	5	4	7	1	3	8	7	4	4	3
F1	B2	5	26	4	21	5	51	4	1	4	1	3	16	7	1	2	8
F1	B2	6	1	5	9	6	6	5	12	5	1	4	2	8	1	3	3
D7	B20	4	16	3	1	4	18	3	1	3	1	0	0	7	1	0	0
D7	B20	5	2	4	9	5	4	4	14	4	16	0	0	8	1	0	0
E11	B21	6	18	6	10	6	1	4	1	6	25	5	2	6	1	0	0

E11	B21			7	5	7	6	5	25	7	6	6	1	7	1	0	0
B6	B22	7	1	5	12	6	9	5	6	6	11	2	10	7	9	2	13
B6	B22	8	1	6	6	7	2	6	2	7	3	3	1	8	2	3	1
D8	B23	6	29	5	22	7	6	4	21	7	10	0	0	7	28	0	0
D8	B23	7	4	6	6	8	4	5	3	8	1	0	0	8	2	0	0
C7	B24	0	0	0	0	0	0	0	0	0	0	0	0	4	1	0	0
C7	B24	0	0	0	0	0	0	0	0	0	0	0	0	6	1	0	0
A9	B25	6	5	6	1	6	1	4	1	4	1	2	1	6	1	0	0
A9	B25	7	3	7	1	7	1	5	1	5	1	3	1	7	1	0	0
G12	B3	5	18	5	8	5	22	4	34	5	28	3	12	6	40	2	1
G12	B3	6	2	6	8	6	4	5	11	6	8	4	1	7	12	3	1
B1	B4	6	7	6	1	7	2	5	2	8	1	3	1	7	5		
B1	B4	5	35	5	5	6	18	4	21	7	2	2	3	6	23	2	3
E7	B5	5	16	3	1	5	24	2	20	6	10	0	0	7	7	0	0
E7	B5	6	3	4	8	6	3	3	3	7	1	0	0	8	3	0	0
B5	B6	6	5	5	17	6	7	5	16	6	4	4	30	7	2	4	15
B5	B6	7	1	6	2	7	1	6	2	7	1	5	6	8	1	5	2
A1	B7	6	1	6	6	7	10	5	5	7	5	2	1	7	1	2	8
A1	B7	7	4	7	2	8	3	6	2	8	1	3	8	8	19	4	2
A10	B8	6	18	6	14	6	12	5	25	6	7	4	3	7	11	2	7
A10	B8	7	2	7	1	7	2	6	3	7	1	5	1	8	1	3	2
B2	B9	5	2	3	3	5	1	2	2	5	1	0	0	7	1	0	0
B2	B9	6	1	4	1	6	1			6	1	0	0	8	1	0	0
H2	C1	6	39	5	35	6	1	5	5	6	1	2	1	7	27	0	0
H2	C1	7	5	6	4	7	12	6	1	7	5			8	10	0	0
C6	C10	6	8	5	6	6	10	4	9	6	14	2	1	7	16	0	0
C6	C10	7	6	6	2	7	1	5	4	7	2			8	4	0	0
D12	C11	5	7	3	14	6	5	2	8	5	11	0	0	6	11	0	0
D12	C11	6	1	4	3	7	1			6	2	0	0	7	1	0	0
F9	C12	7	2	7	1	8	1	5	1	7	1	3	1	8	1	0	0
F9	C12	6	1	6	9	7	2	4	1	6	1	2	3	7	1	2	1
D11	C13	8	2	7	1	8	1	4	9	8	1	0	0	8	5	0	0
D11	C13	7	6	6	2	7	6	3	1	7	6	0	0	7	1	2	1
A11	C14	4	9	2	13	4	20	3	1	5	5	2	9	6	3	0	0

A11	C14	5	1	3	2	5	1	4	2	6	1	3	6	7	1	0	0
C11	C15	6	27	7	5	6	1	4	1	6	1	2	7	7	19	0	0
C11	C15	7	4	8	1	7	6	5	9	7	5	3	1	8	2	0	0
E1	C16	6	5	5	7	6	6	4	1	7	1	3	9	6	13	3	8
E1	C16	7	1	6	2	7	2	5	7	8	1	4	2	7	5	4	2
E3	C17	0	0	0	0	4	1	0	0	0	0	0	0	8	3		
E3	C17	0	0	0	0	3	2	0	0	0	0	0	0	7	7	2	1
E10	C18	6	9	5	1	5	43	4	1	6	12	3	12	7	7	2	16
E10	C18	7	1	6	3	6	9	5	2	7	2	4	2	8	1	3	3
A6	C19	4	18	3	1	4	24	3	1	4	5	0	0	5	2	0	0
A6	C19	5	9	4	7	5	2	4	8	5	1	0	0	6	2	0	0
G7	C2	6	7	5	13	6	12	3	27	6	6	0	0	7	17	0	0
G7	C2	7	1	6	2	7	1	4	4	7	1	0	0	8	3	0	0
E4	C20	6	9	4	31	4	6	2	8	4	22	2	1	6	1	3	13
E4	C20	7	1	5	5	5	1	3	2	5	1	3	3	7	1	4	2
C8	C3	6	4	4	14	5	19	2	1	6	2	2	2	6	10	0	0
C8	C3	7	3	5	2	6	5	3	3	7	1			7	2	0	0
D3	C4	6	10	5	19	6	21	3	1	6	19	3	4	7	6	2	6
D3	C4	7	1	6	5	7	1	4	10	7	3	4	1	8	1	3	1
D9	C5	6	4	5	19	6	9	4	1	5	1	3	1	7	15	0	0
D9	C5	7	2	6	2	7	2	5	3	6	21	4	1	8	2	0	0
F4	C6	8	1	6	6	7	2	6	1	7	3	3	2	8	1	0	0
F4	C6	7	5	5	1	6	26	5	1	6	11	2	8	7	1	2	1
A12	C7	4	1	0	0	6	2	2	8	5	14	0	0	7	4	0	0
A12	C7	3	11	0	0	7	2	3	2	6	5	0	0	8	3	0	0
A5	C8	7	2	3	3	3	3	4	1	6	7			4	3		
A5	C8	6	3	2	1	2	17	3	1	5	47	2	4	3	1	2	6
D10	C9	5	6	0	0	3	1	0	0	3	1	0	0	4	2	0	0
D10	C9	6	3	0	0	4	12	0	0	4	18	0	0	5	1	0	0
D6	D1	7	1	4	1	5	1	3	1	4	1	2	1	6	1	2	1
D6	D1	8	1	5	1	6	1	4	1	5	1	0	0	7	1	3	1
G4	D10	6	11	6	11	5	1	4	15	5	1	2	4	7	12	0	0
G4	D10	7	2	7	1	6	23	5	1	6	17			8	1	0	0
B11	D11	5	1	3	1	5	1	2	1	5	1	2	1	6	1	0	0

B11	D11	6	1	4	1	6	1	3	1	6	1			7	1	0	0
C10	D12	4	32	4	15	4	33	6	1	5	6	3	14	5	5	0	0
C10	D12	5	3	5	4	5	3	7	1	6	2	4	1	6	1	0	0
B10	D13	5	1	0	0	3	1	2	1	2	1	0	0	6	3	0	0
B10	D13	6	1	0	0	4	9			3	25	0	0	7	1	0	0
C2	D14	6	20	5	9	6	35	3	1	7	6	3	2	7	13	2	17
C2	D14	7	6	6	2	7	5	4	10	8	1	4	1	8	1	3	5
F8	D15	6	1	6	3	5	14	2	1	5	1	0	0	6	1	0	0
F8	D15	7	1	7	1	6	9			6	6	0	0	7	1	0	0
F2	D16	7	2	6	1	8	1	5	1	7	2			8	5		
F2	D16	6	10	5	6	7	1	4	8	6	14	2	3	7	9	2	7
F12	D17	6	34	5	12	7	6	4	9	6	1	2	1	7	27	0	0
F12	D17	7	7	6	1	8	1	5	1	7	3	0	0	8	8	0	0
B7	D18	5	4	7	1	6	3	5	3	6	1	3	2	8	2		
B7	D18	6	2	6	2	5	1	4	1	5	1	2	4	6	4	2	1
G6	D19	5	1	4	1	6	3	2	1	5	1	0	0	6	1	0	0
G6	D19	6	1	5	1	7	1			6	1	0	0	7	1	0	0
D2	D2	6	3	5	6	5	23	3	15	5	1	2	6	6	8	0	0
D2	D2	7	1	6	1	6	4	4	3	6	7			7	6	0	0
E8	D20	5	23	2	1	5	1	0	0	6	11	0	0	7	6	0	0
E8	D20	6	5	3	3	6	4	0	0	7	1	0	0	8	1	0	0
A7	D21			0	0	5	8	2	1	7	1	0	0	6	5	0	0
A7	D21			3	5	6	3	3	1	8	1	0	0	7	3	0	0
E2	D22	5	1	4	1	6	15	4	3	5	1	4	12	7	14	3	22
E2	D22	6	8	5	15	7	1	5	1	6	11	5	5	8	2	4	6
E9	D23	4	8	4	12	3	49	5	2	3	1	3	24	4	9	3	12
E9	D23	5	2	5	1	4	16	6	1	4	8	4	2	5	2	4	1
C12	D24	6	18	0	0	6	1	4	1	7	4	0	0	7	1	0	0
C12	D24	7	4	0	0	7	4	8	1	8	1	0	0	8	1	0	0
G10	D3	7	3	5	41	6	44	4	17	7	10	2	3	7	26	0	0
G10	D3	8	3	6	4	7	7	5	1	8	3	3	1	8	4	0	0
E6	D4	7	2	5	9	6	7	5	4	6	10	2	8	7	8	4	1
E6	D4	8	1	6	3	7	3	6	1	7	4	4	1	8	7	5	1
F7	D6	5	23	5	18	6	2	4	21	5	24	2	1	5	1	2	6

F7	D6	6	4	6	3	7	1	5	2	6	2	3	11	6	15	3	1
G5	D7	6	17	5	32	6	21	4	23	6	18	2	7	6	1	0	0
G5	D7	7	2	6	5	7	3	5	4	7	8	0	0	7	16	0	0
B9	D8	5	12	4	2	5	11	2	14	5	3	0	0	6	8	0	0
B9	D8	6	1	5	1	6	2	3	1	6	1	0	0	7	2	0	0
C1	D9	8	2	6	7	8	1	6	4	8	4			8	3		
C1	D9	7	13	5	1	7	13	5	7	7	13	2	11	7	1	2	8
G9	W3	8	1	7	1	7	2	6	2	8	1	4	8	7	6		
G9	W3	7	2	6	3	6	21	5	11	7	2	3	1	6	38	2	5
C9	W3	7	1	7	2	8	1	6	2	7	2	4	1	8	2		
C9	W3	6	15	6	3	6	15	5	13	6	6	3	25	7	6	2	7
B4	W3	7	3	7	1	7	4	6	1	6	22	4	2	8	6	3	1
B4	W3	6	8	6	9	6	15	5	16	5	1	3	1	7	6	2	5
